# Alpha-gal syndrome initially misdiagnosed as chronic spontaneous urticaria in a pediatric patient: a case report and review of the literature

**DOI:** 10.1186/s13256-022-03718-8

**Published:** 2023-01-08

**Authors:** Felicitas Bellutti Enders, Marius Elkuch, Andreas Wörner, Kathrin Scherer Hofmeier, Karin Hartmann

**Affiliations:** 1 Division of Allergy, University Children’s Hospital Basel, Basel, Switzerland; 2 University Children’s Hospital Basel, Basel, Switzerland; 3 Regional Hospital Aarau, Aarau, Switzerland; 4 Division of Allergy, Department of Dermatology, University Hospital Basel and University of Basel, Basel, Switzerland

**Keywords:** Alpha-gal syndrome, Children, Case report

## Abstract

**Introduction:**

Delayed allergy to red meat, also termed alpha-gal syndrome, is increasingly reported in adults and African communities, while pediatric cases remain rare.

**Case presentation:**

Here, we report on a 7-year-old Caucasian boy presenting with recurrent wheals since the age of 5 years old. Episodes with hives occurred around every 3 weeks, mainly in the evening. One of these episodes was also associated with angioedema. No clear trigger was identified. At the first visit, after excluding an infection and autoimmune thyroiditis, chronic spontaneous urticaria was suspected and symptomatic treatment with antihistamines was prescribed. Six months later, the boy presented at the emergency room with generalized urticaria, dyspnoea, and emesis. Symptoms resolved after administration of epinephrine and antihistamines. A detailed medical history after this event revealed that he had eaten three sausages as well as jelly beans containing gelatine several hours prior to this episode. More precisely, after eating the sausages and jelly beans during the day, he had shown some hives before going to bed, and later developed the other symptoms in the middle of the night, suggesting alpha-gal syndrome. In his history, several tick bites are reported. Immunoglobulin E levels for alpha-gal were clearly elevated, confirming the diagnosis of a delayed-appearing immunoglobulin E-mediated allergic reaction to alpha-gal. Emergency medication was prescribed and avoidance of red meat and gelatine-containing foods was recommended. Under this exclusion diet, the boy remained asymptomatic, with the exception of two accidents in the follow up of 3 years, one developing during a barbecue and the second after exceptionally eating marshmallows.

**Conclusion:**

A detailed clinical history led to the diagnosis of alpha-gal syndrome. Although alpha-gal syndrome is typically seen in adults, our case illustrates that children can also present with this potentially life-threatening allergy. Since alpha-gal syndrome is rare in Europe, the disease is not well known and often overlooked for several years, especially in children.

## Introduction

Galactose‐alpha‐1,3‐galactose (alpha‐gal) has recently been identified as a novel food allergen, with a first description in 2008 when immunoglobulin (Ig)E to alpha-gal was identified [1]. Typically, alpha-gal-containing foods, such as red meat and gelatine, are associated with delayed anaphylaxis in sensitized patients, 2–6 hours after ingestion. Symptoms can include urticaria, angioedema, respiratory distress, gastrointestinal symptoms (cramping, nausea, emesis), and also syncope without any other clinical manifestations. Due to the temporal delay of the symptoms, the diagnosis often remains challenging. A detailed medical history may help to identify the allergen and direct the appropriate exclusion diet. Interestingly, this allergen is a carbohydrate expressed in different tissues and cells of mammalians, except in primates. Therefore, the avoidance of red meat and its derivatives (including gelatine-containing sweets) is the mainstay of treatment.

Furthermore, tick bites have been related to the development of this food allergy. In Europe, the most frequent tick is *Ixodes ricinus* and, as in other types of ticks, alpha-gal has been identified in the gut of these ticks [2]. There are three hypotheses that attempt to explain the relationship between tick bites and alpha-gal syndrome by suggesting the tick bite contribute to sensitization: (1) presence of alpha-gal in the saliva of the ticks; (2) presence of mammalian blood from previous bite(s) in the saliva of the ticks; and (3) presence of commensal bacteria expressing alpha-gal, such as *Borrelia burgdorferi*, in the saliva of the ticks [1].

The incidence rates of this allergy are not yet clear, and only few pediatric cases have been described (Table 1), especially in the USA, where meat allergy in general is more frequent [3, 4]. There is only one case series that also describes a pediatric case in Europe [5]. In addition, in a large study on the prevalence of alpha-gal sensitization in Germany, including 107 children out of a total of 1369 patients, the authors did not find a confirmed pediatric alpha-gal syndrome with clinical symptoms, despite a similar prevalence of alpha-gal sensitization between adults and children [6]. With this case report, we want to raise awareness in populations with a low incidence of alpha-gal syndrome, especially in children, where the exposure risk to gelatine-containing sweets might be different than in adults.

**Table 1:** Summary of articles describing children with alpha-gal syndrome

	Wilson *et al*. [3, 4]	Bircher *et al*. [5]	Fischer *et al*. [6]
Number of children included	35	1	107
Study country	USA	Switzerland	Germany
Symptoms			No symptoms associated with alpha-gal sensitization
Hives	31/35 (89%)	1/1 (100%)	NR
Anaphylaxis	17/35 (49%)	1/1 (100%)	NR
Gastrointestinal	23/35(66%)	1/1 (100%)	NR
History of asthma	10/35 (29%)	0/1 (0%)	NR
Tick bite in the last 10 years	35/35 (100%)	NR	NR
Αlpha-gal sIgE > 0.35 IU/mL	34/35 (97%)	1/1 (100%)	ND^a^
Positive basophil activation tests	ND	1/1 (100%)	ND

*NR* not reported, *ND* not determined

^a^11% for total cohort

## Case presentation

Our patient started to develop wheals every couple of weeks at the age of 5 years old. After 2 years of evolution, a first diagnostic workup could not identify a clear trigger of the hives. Skin prick testing for inhalative allergens remained negative for different pollen, house dust mites, mold, and animal dander. Laboratory investigations showed that total IgE levels (22 IU/mL) and tryptase (4.7 ng/mL) were within normal ranges, thyroid function was adequate, no anti-thyroid peroxidase (TPO) antibodies were detected, and there were no signs of infection with parasites or *Helicobacter pylori*. The patient was discharged with the working hypothesis of chronic spontaneous urticaria, and antihistamines were prescribed for symptomatic treatment.

Six months later, the boy presented at the emergency room during the night with generalized urticaria, edema, dyspnoea, and vomiting. Symptoms resolved after administration of emergency treatment, including epinephrine injector. Tryptase levels were clearly elevated (44.2 ng/mL). Several hours prior to these symptoms, earlier that same day, he had eaten three Cervelat sausages (typical Swiss sausages containing beef and pork meat) as well as jelly beans containing gelatine, had already shown some wheals before going to bed, and then developed the other symptoms in the middle of the night, suggesting a delayed allergic reaction to meat. The mother also reported that he has had several tick bites in the past and that the only meat he eats regularly is the Cervelat sausage. The level of specific IgEs for alpha-gal was elevated with 21.2 kUA/L, levels for gelatin (bovine) were < 0.1kU/L, whereas tryptase levels had normalized after the reaction (6 ng/mL), which confirmed the suspicion of a delayed-appearing IgE-mediated allergic reaction to meat. Emergency medication was prescribed as well as avoidance of red meat and other gelatine-containing foods, and a treatment plan was provided. Under the avoidance diet, all symptoms including wheals resolved—together with the laboratory results, this again confirmed the diagnosis of alpha-gal syndrome. Over the last 3 years, the family reported two accidents, one after eating a red meat-containing sausage at a barbecue (BBQ) and one after ingestion of marshmallows. Both accidents happened at school and the patient only presented with hives. As suggested by Mullins *et al*. in 2012, alpha gal might be the target of reactivity to gelatin, explaining the reaction after the second accidental ingestion, although the IgE levels for gelatin were not elevated [7].

This observation also indicates that the severe reaction 3 years ago, which had required emergency treatment with administration of epinephrine, was probably caused by the large amount of ingested allergen at that time. Coexistence of chronic spontaneous urticaria and alpha-gal syndrome is theoretically possible; however, complete resolution of the symptoms under avoidance therapy and recurrence of urticaria after accidental ingestion make it rather unlikely that the patient ever presented a chronic spontaneous urticaria.

## Discussion and conclusion

Our case underscores again that diagnosis of alpha-gal syndrome can be challenging, mainly because of the temporal delay between ingestion of the allergen and manifestation of symptoms, and especially in children, where the disease remains rare. Furthermore, our case shows that alpha-gal syndrome can also present with atypical symptoms. Especially in children, the ingestion of gelatine-containing sweets is often overlooked by parents/caretakers as it is not considered as an allergen. Therapy of alpha-gal syndrome relies on avoidance of alpha gal-containing foods. To date, no tolerance induction has been suggested for this delayed-appearing IgE-mediated allergic reaction.

## Data Availability

Not applicable.
